# Molecular basis of post-translational neoantigen generation in rheumatoid arthritis: breaking immunological tolerance and implications for targeted therapy

**DOI:** 10.3389/fimmu.2026.1876035

**Published:** 2026-08-20

**Authors:** Alba Pérez Linaza, Sussel Picallo Díaz, Isabel Serrano García, Wenjie Yi, Álvaro Guerrero Lores, Juan Manuel Aragón González, Francisco Garcia-Cozar, Cecilia Fernandez-Ponce, Ricardo Fernandez-Cisnal

**Affiliations:** 1 Rheumatology Service, Puerta del Mar University Hospital, Cádiz, Spain; 2 Biomedical Research and Innovation Institute of Cadiz (INiBICA), Research Unit, Puerta del Mar University Hospital, Cádiz, Spain; 3 Department of Biomedicine, Biotechnology and Public Health, School of Medicine, University of Cádiz, Cádiz, Spain; 4 Cardiology Service, Virgen del Rocío University Hospital, Seville, Spain

**Keywords:** autoimmune diseases, immunological tolerance, neoantigen, post-translational modifications, rheumatoid arthritis, therapeutic targets

## Abstract

Autoimmune diseases are characterized by a loss of immune system tolerance to self-antigens, which can trigger an immune response capable of affecting various organs and tissues. An increase in the prevalence of these diseases has been reported in recent years. Rheumatoid arthritis (RA) is a chronic, systemic autoimmune disorder characterized by persistent synovial inflammation. While it predominantly targets small and medium-sized joints, its pathogenesis frequently extends to diverse extra-articular manifestations. In line with other autoimmune diseases, the presence of neoantigens derived from post-translational modifications represents a critical checkpoint in RA pathogenesis, driving the loss of self-tolerance and the subsequent perpetuation of the autoreactive immune response. The primary therapeutic goal in RA is to achieve clinical remission or, alternatively, to maintain low disease activity. Therapeutic strategies designed to reach these targets are currently available in clinical practice. However, most of these agents act systemically, potentially leading to significant adverse effects. This review article explores the molecular landscape of post-translational modifications and analyzes how these chemical alterations generate neoantigens, which contribute to the pathogenesis of RA. Furthermore, the identification of these specific molecular signatures offers a pathway toward precision medicine, moving beyond conventional treatments to targeted therapies that could potentially restore tolerance, improving patient quality of life and clinical outcomes.

## 1 Introduction

Autoimmune diseases (ADs) are a group of conditions characterized by a loss of immune system tolerance to the individual’s own antigens (Ags), triggering specific immune responses against self-antigens (self-Ags), which can cause damage to different organs and systems (1). Although the real incidence of ADs is difficult to ascertain, estimates suggest that they may affect approximately 10% of the global population. With their prevalence having increased in recent years in industrialized countries, they have emerged as a major cause of morbidity and mortality. They affect women more often, and although they can develop at any age, some occur within specific age ranges, as is the case of rheumatoid arthritis (RA) and multiple sclerosis. The etiopathogenesis of ADs is very complex and not yet fully understood. Some individuals are genetically predisposed, or susceptible, and are affected by a series of environmental factors, including sex hormones, infections, and toxic agents, that favor the development of the disease (2, 3).

RA is a chronic, systemic autoimmune inflammatory disease characterized primarily by proliferation of the synovial membrane of the joints, with pannus formation and a tendency to destroy the articular cartilage. Joint involvement is mainly symmetrical in small and medium-sized joints, particularly those of the hands and feet. Additionally, extra-articular manifestations and systemic involvement may arise at any stage of the disease. The prevalence of RA ranges from 0.5% to 1%, and, as in other ADs, its incidence is higher in women (4, 5).

**Figure 1:**
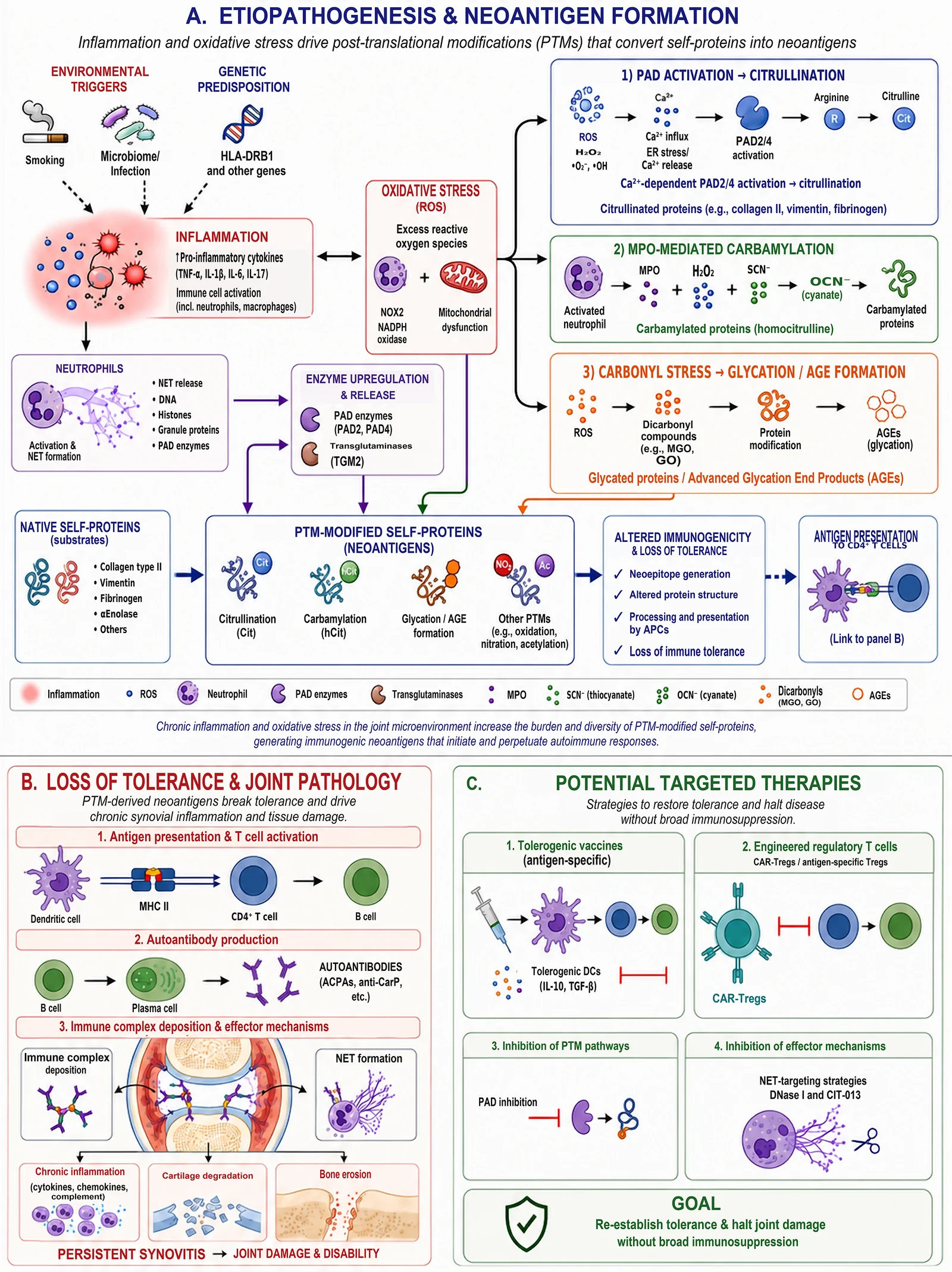
From post-translational modification of self-proteins to immune tolerance breakdown and emerging targeted therapies in rheumatoid arthritis. **(A)** Chronic inflammation and oxidative stress, driven by environmental factors (e.g., smoking and microbiome dysbiosis or infection) acting on a genetically susceptible background (e.g., *HLA-DRB1*), promote the generation of reactive oxygen species (ROS), neutrophil activation and neutrophil extracellular trap (NET) formation, together with the upregulation of protein-modifying enzymes such as peptidylarginine deiminases (PADs) and transglutaminases. These processes induce multiple post-translational modifications (PTMs), including citrullination, carbamylation and glycation/advanced glycation end product (AGE) formation, generating modified self-proteins with increased immunogenicity. The resulting neoepitopes are more efficiently processed and presented by antigen-presenting cells (APCs), ultimately contributing to the breakdown of immune tolerance. **(B)** PTM-derived neoantigens are presented to autoreactive CD4^+^ T cells, promoting B-cell activation and differentiation into autoantibody-producing plasma cells, including anti-citrullinated protein antibodies (ACPAs) and anti-carbamylated protein (anti-CarP) antibodies. Immune complex deposition, cytokine production, complement activation and NET formation amplify chronic synovial inflammation, ultimately leading to cartilage destruction, bone erosion and persistent joint pathology. **(C)** Emerging therapeutic strategies aim to interrupt this pathogenic cascade by restoring antigen-specific immune tolerance or preventing PTM generation and downstream inflammatory mechanisms. These approaches include antigen-specific tolerogenic vaccines, tolerogenic dendritic cells, CAR-Tregs and antigen-specific regulatory T cells, PAD inhibitors, and NET-targeting strategies (e.g., DNase I or CIT-013), with the goal of restoring immune tolerance, limiting chronic synovial inflammation and preventing progressive joint damage while minimizing systemic immunosuppression.

It has been proposed that various genetic and environmental factors participate in the etiology of RA, in such a way that genetically predisposed or susceptible individuals are exposed to a series of environmental factors leading to an abnormal activation of the immune system characterized by a loss of tolerance to autoantigens, which trigger autoimmune responses. Thus, genetic susceptibility factors, such as the HLA system, non-HLA genes, and other epigenetic mechanisms, are not sufficient on their own to trigger the onset and development of the disease, although they may play an important role in pathogenesis, progression, and response to treatment (**Figure 1A**) (4–6).

According to the pathogenesis of RA, in the preclinical stage of RA, different risk factors and the development of alterations of the immune system take place before joint involvement occurs. In genetically predisposed individuals, exposure to one or more environmental factors leads to post-translational modifications of self-antigens, such as citrullination, carbamylation or acetylation of proteins located in synovial membrane, and other tissues, including the lung or periodontal mucosa. These modified proteins act as neoantigens, which are recognized by the immune system and presented to CD4+ T lymphocytes via MHC class II molecules. This interaction stimulates B lymphocytes to synthesize autoantibodies. Furthermore, other stimuli such as the formation of immune complexes, alterations in microcirculation, or activation of the complement system promote cellular migration to the affected tissues and the development of synovitis (**Figures 1A, B**) (7–9).

Some important pathogenic changes occur in the synovial membrane of patients with RA. One of them is the expansion of the intima lining due to the proliferation and activation of synoviocytes, specifically macrophage-like synoviocytes (MLSs) and fibroblast-like synoviocytes (FLSs). While MLSs serve as a primary source of pro-inflammatory cytokines, FLSs are characterized by the secretion of matrix metalloproteinases and small-molecule mediators like prostaglandins and leukotrienes. This activated FLS phenotype is further driven by specific microRNA expression patterns. Consequently, FLSs adopt an invasive behavior capable of migrating between joints to propagate the disease, while actively supporting ectopic lymphoid structure formation through interactions with adaptive immune cells. On the other hand, the synovial sublining exhibits infiltration by adaptive immune cells, especially CD4+ T lymphocytes, which either diffusely populate the tissue or aggregate into ectopic germinal centers in 15–20% of patients. Within these structures, mature B cells and plasma cells undergo local affinity maturation suggesting an immune response against native or altered peptides (**Figure 1B**) (8, 9).

It’s critical to note that the chronic inflammatory milieu characteristic of RA promotes dysfunction of regulatory T cells (Tregs) and the loss of their suppressive capacity, which prevents effective control of autoreactive T cells and favors the differentiation of Tregs into pathogenic Th17 cells (8–10). In addition, macrophages, follicular dendritic cells and mast cells are widely distributed throughout the synovial sublining. Thus, this cellular infiltration and its immune activity drive the formation of an inflammatory tissue called *pannus* at the interface between cartilage and bone, triggering progressive joint erosion and bone destruction (**Figure 1B**) (8, 9, 11).

Unlike normal immune responses, complete resolution does not occur due to the existence of mechanisms that amplify the immune response. Furthermore, tissue damage and alterations in protein structure can result in the emergence of new epitopes, different from the original epitope that triggered the immune response, that are targeted by specific lymphocytes, promoting the exacerbation and chronicity of the disease. This phenomenon is known as epitope spreading and is crucial in perpetuating the pathology (12–14).

In summary, the pathogenesis of this disease is due to the loss of Treg cells suppressive capacity in the affected tissues and the existence of a pro-inflammatory vicious circle caused by the presentation of antigens to B cells, which produce autoantibodies, generating immune complexes and perpetuating inflammation. This chronic inflammatory environment is sustained by the immune cells mentioned above, autoantibodies, cytokines and other inflammatory mediators, leading to progressive joint destruction (**Figure 1B**).

## 2 Neoantigens in RA

In line with the aforementioned immunopathogenesis of RA, the generation of neoantigens plays an important role (6, 14). *Neoantigens* are self-Ags that undergo changes in their original structure and can trigger an immune response when they are recognized as foreign by lymphocytes, thus breaking immune tolerance (15, 16).

Among the potential mechanisms involved in the generation of neoantigens are epigenetic changes, epitopes generated by point mutations, alternative RNA splicing, defective ribosomal products (DRiPs) or aberrant post-translational modifications (16–18). Despite being mechanistically distinct, these processes share a common outcome: the diversification of the cellular proteome beyond the canonical genetic sequence, resulting in the generation of previously unrecognized peptides that can be processed and presented by MHC molecules. Although the available evidence indicates that these mechanisms generate transcriptomic and proteomic diversity and may serve as potential sources of neoepitopes, their contribution to autoimmunity in RA, like in other ADs, is still an area of active research (19, 20).

Regarding the generation of neoantigens by post-translational modifications (PTMs), these are chemical changes, either spontaneous or enzyme-mediated, occurring in the structure of proteins after their synthesis. Although these changes are part of normal physiology, they can also occur under inflammatory or oxidative stress conditions, generating modified self-proteins with altered immunogenicity that may trigger an immune response (15–17, 21) (**Figure 1A**).

While the immune system employs several mechanisms to eliminate autoreactive lymphocytes, in the periphery, PTMs, driven by inflammation or oxidative stress, can generate novel proteoforms that are absent during central tolerance induction in primary lymphoid organs. Consequently, these modified self-Ags can be recognize as “nonself”, triggering both T and B cell-mediated autoimmune response (22, 23). While T cell-mediated immune responses to PTM-generated neoantigens tend to be specific to the modified peptides and do not recognize unmodified self-peptides, B cells can release antibodies (Abs) that can recognize both modified and unmodified antigens (24, 25). Furthermore, autoimmune responses triggered by self-Ags that have undergone PTMs may subsequently spread to other intra- and intermolecular determinants (epitope spreading), favoring the chronic course of the disease (23, 26).

PTM-modified self-Ags have been identified in many ADs. One such example is RA, in which the occurrence of various modifications associated with different self-Ags has also been described (22, 23, 27–29).

Thus, PTM-derived neoantigens are pivotal in the pathogenesis of ADs like RA and represent potential indicators for assessing disease progression (15, 23, 30). A deeper insight into the nature and origin of these neoantigens could enhance diagnostic precision and promote the development of potential targeted therapies in RA and in other ADs, offering a more selective mechanism of action minimizing the systemic adverse effects associated with current treatments (15, 24).

The following sections examine PTM-derived neoantigens in RA. **Table 1** details neoantigens generated by some of the PTMs discussed in this review, as well as the species and the biological sources where these neoantigens have been detected, and the methods used for their detection. Studies based solely on serological reactivity without direct antigen detection, or those reporting PTMs outside the predefined scope, were excluded. Due to the vast number of citrullinated proteins identified in RA, only the most well-characterized neoantigens with direct experimental evidence are listed.

**Table 1:** Landscape of PTM-derived neoantigens and their tissue distribution in RA.

Protein	Tissue/biofluid (source)	Species	Evidence type	PTM detected	Amino acid sites where protein modification occur	Method
β-actin	Synovial fluid (inflamed joint)	Human	Direct	Citrullinated β-actin	Arg196 Arg206 Arg210	LC–MS/MS citrullinome (54)
A1AT	Serum	Human	Direct	Carbamylated A1AT	Lys125	Affinity chromatography - LC-MS/MS and ELISA (95)
	Synovial fluid (inflamed joint; RA)	Human	Direct (MS)	Carbamylated A1AT	Lys125	Affinity chromatography - LC-MS/MS and ELISA (95)
Albumin	Serum	Human	Direct	Carbamylated albumin	N/A	LC–MS/MS (91)
	Joint tissues (cartilage/synovial tissue/synovial fluid)	Human	Direct	Carbamylated albumin peptides	K257 K402 K438	Whole-proteome LC–MS/MS carbamylome (92)
apoE	Synovial fluid (inflamed joint)	Human	Direct	Citrullinated apoE	Arg198	LC–MS/MS citrullinome (54)
COL2	Joint cartilage	Human/Rat	Indirect	Glycosylated and de-glycosylated immunodominant COL2	Lys264	Protein purification from tissue samples followed by T-cell hybridoma assays (40)
	Joint tissues (cartilage/synovial tissue/synovial fluid)	Human	Direct	Carbamylated collagen peptides	K250 K620 K773 K929	Whole-proteome LC–MS/MS carbamylome (92)
Fibrinogen	Synovial fluid	Human	Indirect	Citrullinated fibrinogen	N/A	Electrophoresis/ Western blot analysis/MALDI-TOF-MS (55)
Fibronectin	Synovial fluid	Human	Indirect	Citrullinated fibronectin	N/A	Electrophoresis/ Western blot analysis/MALDI-TOF-MS (55)
	Joint tissues (cartilage/synovial tissue/synovial fluid)	Human	Direct	Carbamylated fibronectin peptides	K486 K987 K1050	Whole-proteome LC–MS/MS carbamylome (92)
IgG	Serum	Human	Direct	Aberrant IgG galactosylation pattern	Asn297	HPLC-based N-glycan quantification (35)
	Serum (prior to the onset of RA)	Human	Direct	Aberrant ACPA/IgG1 galactosylation and fucosylation pattern	Asn297	Nano-LC-MS (36)
	Serum	Human	Direct	Aberrant IgG galactosylation and sialyation pattern	Asn297	MALDI-TOF MS (37)
	Plasma and synovial fluid	Human	Direct	ACPA IgG - Fc glycosylation	N/A	LC-MS (38)
	Serum (RA and other inflammatory arthropathies)	Human	Indirect	IgG modified by AGE	N/A	ELISA (49)
	Serum immune complexes / serum	Human	Indirect	AGE-modified IgG	N/A	PEG precipitation of immune complexes - Affinity Chromatography – immunoblotting (50)
MNDA	Synovial fluid (inflamed joint)	Human	Direct (MS)	Citrullinated MNDA	Arg127 Arg129	LC–MS/MS citrullinome (54)
Vimentin	Synovial fluid	Human	Direct	Citrullinated vimentin	N/A	Electrophoresis/ Western blot analysis/MALDI-TOF MS (55)
	Joint tissues (cartilage/synovial tissue/synovial fluid)	Human	Direct	Carbamylated vimentin	K235 K448	Whole-proteome LC–MS/MS carbamylome (92)

A1AT, Alpha-1 antitrypsin; ACPA, Anti-citrullinated protein antibodies; AGE, Advanced glycation end-products; anti-CarP, Antibodies directed against carbamylated proteins; apoE, Apolipoprotein E; COL2, Collagen type II; ELISA, Enzyme linked immunosorbent assay; IgG, Immunoglobulin G; LC-MS/MS, Liquid chromatography–mass spectrometry/ mass spectrometry; Nano-LC-MS, Nano-liquid chromatography mass spectrometry; MNDA, Myeloid cell nuclear differentiation antigen; MS, Mass spectrometry; RA, rheumatoid arthritis; N/A, Not available due to lack of specific modification site data in current literature.

## 3 Post-translational modifications as a mechanism of neoantigen generation in RA

PTMs are chemical changes that occur in the structure of proteins after their synthesis, giving rise to neoantigens that can alter immune tolerance and favor the development of ADs, including RA (15, 31).

Selected examples of PTMs and their role in neoantigen generation in RA are summarized in **Figure 1A** and detailed below.

### 3.1 Glycosylation

*Glycosylation* consists of the covalent attachment of carbohydrate moieties to either a nitrogen (N-glycosylation) or an oxygen (O-glycosylation) atom within the amino acid side chains of a protein. This process plays a role in epitope presentation and in the regulation of the immune response mediated by immunoglobulins (Igs). When glycosylation occurs aberrantly, it can alter antigen presentation, inducing a loss of immune tolerance, and can also modify the properties of Igs, thereby affecting the immune response (32, 33).

In patients with RA, aberrant glycosylation of IgG acts as a potent immunogenic stimulus (34, 35) and could be part of the preclinical processes that precipitate the onset of RA, thus preceding diagnosis (35). In addition, IgG1 anti-citrullinated protein antibodies (ACPAs) may exhibit significant alterations in Fc galactosylation and fucosylation before RA onset, potentially driving a pro-inflammatory phenotype (36). Notably, these modifications also correlate with pregnancy-induced clinical improvement of RA and postpartum relapse (37) (**Table 1**).

Blöchl et al. observed that Fc-region proteoforms of IgG antibodies, especially ACPA, distinguish autoimmune responses in patients with RA between plasma and synovial fluid, highlighting that immune activity is compartmentalized. They also found that certain structural modifications of these antibodies (such as changes in glycosylation or subclasses) correlate with disease activity. Future clinical research are needed to better understand the role of autoantibody proteoforms in shaping immune responses (38) (**Table 1**).

Another self-Ag capable of triggering an immune response in RA is collagen type 2 (COL2) (39). The immunodominant COL2 epitope is glycosylated at K264 in the articular cartilage of healthy individuals, while both glycosylated and non-glycosylated forms coexist in the cartilage of RA patients (39, 40) (**Table 1**). This heterogeneity elicits a robust COL2-specific T cell reactivity against both proteoforms, thereby driving the inflammatory response and contribuiting to the development and perpetuation of the disease (39, 41). According to these findings, Michaëlsson et al. evidenced that mice immunized with carbohydrate-depleted COL2 developed a less aggressive form of arthritis compared to the standard glycosylated collagen-induced arthritis (CIA). This effect is thought to be driven by T-cell-specific recognition of the glycosylated epitope, particularly since the overall COL2-specific antibody response remains unaffected by the loss of these carbohydrate moieties (39, 42).

### 3.2 Glycation

*Glycation* refers to the modification of protein amino groups by reducing sugars. Advanced glycation end-products (AGEs) are compounds formed through the non-enzymatic interaction of reducing sugars and proteins, nucleic acids, or lipids, and are known to alter the original structure of proteins and activate the receptor for advanced glycation end-products (RAGE) (43). AGEs can bind to RAGE, triggering an inflammatory response and activity (43, 44). Oxidative stress under chronic inflammation has also been identified as a key driver of AGE formation (45). Furthermore, RAGE has been implicated in the pathogenesis of various ADs, including RA (43).

Knani et al. found that serum levels of AGEs, specifically N^ϵ^-carboxymethyllysine (CML) and pentosidine, were significantly elevated in patients with RA compared to controls. Moreover, AGE concentrations were higher in patients with greater disease activity, and increased CML levels were significantly associated with RA severity (46). Similar findings were reported for methylglyoxal (MGO) (47). Higher plasma concentrations of transthyretin (TTR) and RAGE have also been found in patients with RA, a positive correlation that may be pathologically significant, as their interaction may activate inflammatory cascades (48). Furthermore, stimulation of the NF-κB pathway by tumor necrosis factor α (TNF-α) appears to be essential for the deregulation of TTR and RAGE expression (48).

Under conditions of hyperglycemia and/or oxidative stress, IgG is another protein whose primary structure can be altered by AGEs. AutoAbs to IgG-AGE have been detected in patients with severe and long-standing RA, but while IgG-AGE has been found in early onset RA, the production of anti-IgG-AGE antibodies appears restricted to a specific subgroup of RF-seropositive patients characterized by elevated disease activity (49). The presence of these autoAbs has been associated with higher levels of immune complexes in blood, which could contribute to the development or worsening of the disease. AGE-modified proteins and anti-AGE immune response could act as important diagnostic and prognostic markers of RA (50) (**Table 1**).

### 3.3 Citrullination

*Citrullination* is a modification in which the amino acid arginine is converted into citrulline, in a process catalyzed by calcium-dependent peptidylarginine deiminases (PADs) (51–53).

Citrullination is involved in normal physiological processes, such as apoptosis, regulation of gene expression, embryonic development, and neutrophil extracellular traps (NETs) activation and release (NETosis) (51, 52) (**Figure 1B**). Numerous citrullinated cellular and extracellular proteins have been described in the synovial tissue of patients with RA, including vimentin, α-enolase, antithrombin, COL2, fibrin, fibrinogen, fibronectin, β-actin, apolipoprotein E (apoE), myeloid cell nuclear differentiation antigen (MNDA) and arginine, which can give rise to neoantigens recognized by ACPAs and trigger a specific T-cell response (31, 39, 53–55) (**Figures 1A, B**) (**Table 1**).

Importantly, in RA, ACPA synthesis is strongly linked to the *shared epitope* phenomenon (53, 56).

Citrullination can also occur in inflamed tissues beyond the joints and in other inflammatory conditions. In recent years, the role of mucosal sites in the pathophysiology of RA has gained significant attention (**Table 2**). In the oral mucosa, periodontitis, driving mainly by *P. gingivalis* and *A. actinomycetemcomitans*, can promote protein citrullination, thereby generating neoantigens capable of triggering autoimmune responses (53, 57–60). Moreover, the presence of Abs directed against carbamylated and acetylated proteins has also been described (61, 62). In the lungs, exposure to tobacco smoke and inhaled particles, such as silica, can promote local inflammation and citrullination. Consequently, the pulmonary mucosa may represent an early site of RA-autoantibody production (53, 57). Finally, while the gut microbiota is known to modulate the host immune system in various autoimmune and inflammatory diseases, this relationship is further evidenced in RA by the presence of gut dysbiosis, which suggests that the intestinal mucosa might also contribute to the generation of modified antigens (61–63) (**Table 2**).

**Table 2:** The role of mucosal tissues in the pathophysiology of RA.

Mucosal tissue	PTMs	Source of PTMs
Oral	Citrullination (53, 57–60)	- Periodontal disease: *P. gingivalis* and *A. actinomycetemcomitans* are the two most important microorganisms involved in the pathogenesis of periodontal disease and RA. - Increase PAD activity (*P. gingivalis*). - Production of ACPA.
	Carbamylation and acetylation	- Anti-CarP and AAPAs can be secreted locally in the oral mucosa (61, 62).
Lung	Citrullination (53, 57)	- Exposure to tobacco smoke and other nanomaterials (such as silica dust) can provoke inflammation and citrullination in the lungs, even in the absence of other manifestations of RA. - Hypothesis: the lungs may be the initial site of RA-autoantibody production. - Protein citrullination in the lungs may contribute to autoimmunogenicity of RA and generate ACPA.
	Redox	- Chronic inflammation characteristic of RA promotes oxidative stress and AGE formation (43, 118, 122).
	Carbamylation	- Carbamylated proteins have been demonstrated in the lungs of patients with chronic obstructive pulmonary disease (62). - Anti-CarP have been strongly associated with ILD (96).
Intestinal	Citrullination, carbamylation and acetylation	- Changes in the composition of normal microbiota (dysbiosis) may disrupt immune system function. - Microbiota of RA patients has been described as potentially altered (63). - Intestinal mucosa might also play a role in the pathophysiology of RA (62). - Citrullinated, carbamylated and acetylated proteins may have their origin in the gut. - Further studies are needed to determine the role of the intestinal mucosa in the onset of the AMPA responses in RA (61).

AAPAs, anti-acetylated protein antibodies; ACPA, anti-citrullinated protein antibodies; AGE, advanced glycation end-products; AMPA, anti-modified protein/peptide antibodies; anti-CarP, autoantibodies directed against carbamylated proteins; ILD, interstitial lung disease; PAD, peptidylarginine deiminase enzyme, RA: rheumatoid arthritis.

In RA, citrullination may involve various proteins, primarily endogenous self-Ags located in organs and tissues implicated in the immunopathogenesis of inflammatory disease. The presence of citrullinated COL2 in the articular cartilage and synovial fluid of RA patients, together with Abs to these modified epitopes, can promote the development of immune-mediated arthritis (64–66). Additionally, Snir et al. observed elevated levels of Abs to citrullinated RA-associated Ags, such as vimentin, fibrinogen, α-enolase, and COL2, in patients’ synovial fluid compared to serum (67).

Citrullinated keratin has been also identified as a self-Ag within the synovial tissue of RA-affected joints. Furthermore, serum levels of autoAbs targeting type II hair keratin Hb4 are significantly elevated in RA patients compared with healthy controls, reflecting its role in the inflammatory process (68).

Another citrullinated protein for which autoAbs have been described in RA is the immunodominant citrullinated alpha-enolase peptide 1 (CEP-1), found in synovial fluid (69). In addition, citrullinated glucose-6-phosphate isomerase (70) and citrullinated fibrin (71) may also act as self-Ags in this inflammatory disease.

According to ACPAs, these are directed against citrullinated peptides and exhibit cross-reactivity, enabling recognition of various types of citrullinated proteins, including fibrinogen, myelin basic protein, and collagen (72), as well as peptides that have undergone other PTMs (32). Like RF, ACPAs can be detected in the serum of patients several years before the onset of RA symptoms and can predict its development (73, 74). Some researchers have reported that some RF-negative patients are ACPA- positive, while a higher percentage of RF-positive patients also have the presence of ACPAs, although some variability remains across the literature. Patients positive for both RF and ACPAs are at increased risk of developing RA (75, 76) (**Figure 1B**).

The presence of antibodies to citrullinated proteins as CEP-1, in patients with RA, has correlated with joint erosion and interstitial lung disease (ILD), a significant extra-articular manifestation of RA with high morbidity and mortality (77, 78). Furthermore, the presence of Abs against citrullinated fibrinogen peptides has been associated with lung tissue alterations in patients with untreated early-stage RA (79) (**Table 2**), while Abs against mutated citrullinated vimentin (anti-MCV) and Cyclic Citrullinated Peptide has been linked to rheumatoid nodules and cutaneous vasculitis, among other manifestations (80, 81).

Interestingly, both RA-associated interstitial lung disease and the erosive phenotype of the disease have been closely associated with the presence of antibodies targeting PAD enzymes, particularly those that react specifically with isoform 4 or those exhibiting cross-reactivity with both PAD4 and PAD3 (82–84). Notably, anti-PAD4 autoantibodies, identified by their cross-reactivity with PAD3, increase the catalytic efficiency of PAD4 by lowering its calcium requirement. This unique mechanism, where autoAbs activate enzymes that produce further citrullinated antigens, establishes a self-perpetuating loop that accelerates joint erosive progression (85). Interestingly, elevated levels of citrullinated PAD4 have been detected in the synovial fluid of RA patients relative to osteoarthritis controls. Unlike unmodified PAD4, the autocitrullinated form recruits monocytes *in vitro* and promotes joint inflammation in murine models, indicating that PAD4 autocitrullination is a critical mechanism in the inflammatory process of RA (86).

### 3.4 Carbamylation

*Carbamylation* is characterized by the addition of a cyanate group to the protein structure (33). This PTM occurs at different sites in the body and, like glycation, has been associated with molecular ageing and other pathological processes. In the context of RA, lysine carbamylation is considered one of the most relevant findings. The enzyme lysine carbamyltransferase catalyzes the carbamylation of lysine to homocitrulline (hCit) in a process requiring cyanate (**Figure 1A**) (33, 87). Exposure to tobacco smoke, uremia, and inflammation can elevate cyanate levels and thereby induce carbamylation (87, 88) (**Figure 1A**).

Carbamylation alters the original protein structure and can result in the loss of immune tolerance, leading to the synthesis of autoAbs directed against carbamylated proteins (anti-CarP) (33) (**Figure 1B**). Beyond citrullination, the pathogenesis of RA involves a broader, polyclonal response against multiple modified antigens. This is evidenced by the detection of antibodies against various carbamylated proteins, which are present not only in ACPA-positive cohorts but also in ACPA-negative patients, highlighting carbamylation as a critical factor in the RA autoimmune response (89, 90).

Given that antigens like fibrinogen and vimentin can be targeted by both ACPAs and anti-CarP antibodies, researchers have focused on identifying antigens recognized exclusively by the latter. Carbamylated albumin was the first such antigen identified, not cross-recognized by ACPAs. Nakabo et al. detected carbamylated albumin in the serum of RA patients and observed that individuals with anti-carbamylated albumin antibodies exhibited higher serum myeloperoxidase (MPO) levels. These levels correlated with disease activity, suggesting that MPO released from inflammatory sites drives albumin carbamylation, thereby promoting autoantibody production. Notably, carbamilated albumin, like other proteins such as collagen, fibronectin or vimentin, has also been detected within the joints of RA patients (91, 92) (**Table 1**). Additionally, MPO, known to induce carbamylation of cartilage proteins in inflammatory conditions (39), has been found at significantly higher plasma levels in RA patients compared to healthy controls and those with osteoarthritis (93, 94).

Carbamylated alpha-1 antitrypsin (A1AT) has also been found in the synovial fluid of RA patients and described as a target of anti-CarP antibodies, suggesting that its recognition by these Abs in the joint may contribute to synovial inflammation in anti-CarP-positive RA patients (95) (**Table 1**).

Anti-CarP antibodies have been strongly associated with ILD (96), greater joint damage and radiographic progression, and even in ACPA-negative patients, the presence of anti-CarP antibodies correlates with a more severe course of the disease (97, 98). Despite conflicting evidence, some authors have identified an association between anti-CarP antibodies and higher disease activity as well as greater functional disability at disease onset and during long term follow up (99). Similar to ACPAs and RF, anti-CarP antibodies can be detected years prior to the onset of disease symptoms and can serve as predictors of disease progression in patients presenting arthralgia (100, 101). Interestingly, like ACPAs, anti-CarP antibodies have been associated with endothelial dysfunction in patients with RA, suggesting that they constitute a risk factor for the development of atherosclerosis and the pathogenesis of cardiovascular disease (102–104) (**Figure 1B**).

In general terms, the autoAbs described in RA are known as anti-modified protein/peptide antibodies (AMPAs) and can exhibit cross-reactivity, such that the same Ab can recognize peptides with different PTMs (105). Cross-reactivity between ACPAs and anti-CarP is common in RA patients, as has been observed for the autoAg α-enolase (106), as well as for the citrullinated and carbamylated fibrinogen epitopes (107). Despite the cross-reactivity, anti-CarP has been linked to more severe joint disease and significant extra-articular manifestations and may aid in identifying RA subgroups with poor prognosis, including those who are ACPA-negative cases (97, 98).

The presence of anti-CarP antibodies is not restricted to adults. Juvenile idiopathic arthritis (JIA), the most common chronic inflammatory rheumatic disease in children, includes seven subtypes of arthritis with onset before age 16 and unknown cause (108). As most JIA patients are ACPA-negative, new markers are needed to predict disease severity. Muller et al. detected anti-CarP antibodies in JIA patients, including those negative for both ACPA and IgM-RF, although no association was found with disease progression, age, antinuclear antibodies, or disease activity (109). Further studies are needed to clarify the diagnostic and prognostic value of anti-CarP in JIA.

### 3.5 Acetylation

*Acetylation* is a reversible enzymatic process involving the addition of an acetyl group to the free amines of lysine residues in proteins (105, 110).

Some authors have proposed that, like citrullination and carbamylation, acetylation may be involved in the pathogenesis of RA. Juárez et al. identified anti-acetylated vimentin Abs in the serum of patients with early-stage RA and suggested a possible link between the microbiome, acetylation of self-Ags, and the development of autoimmunity (110).

Anti-acetylated protein antibodies (AAPAs) in RA have been explored as biomarkers and as potential classification tools. Some researchers have found that anti-acetylated ornithine antibodies could enhance RA classification, even though they lacked statistical significance as biomarkers (111). Accordingly, AAPAs have been detected in sera via three vimentin-derived acetylated peptides, in early RA cohorts, including those negative for RF and ACPA. Crucially, the simultaneous triple AAPA positivity further increased diagnostic specificity, bridging the gap in seronegative RA diagnosis (112).

Additionally, García-Moreno et al. developed citrullinated, homocitrullinated, and acetylated chimeric peptides to evaluate anti-AMPAs as biomarkers of ILD and severe RA. Autoantibodies, particularly IgA and IgM isotypes, were associated with more aggressive disease, while IgG against the triple-modified peptide correlated with severe joint damage and higher titers in ILD patients (105).

Although further studies are needed to fully understand the role of acetylation and AAPAs in RA pathogenesis. These findings may also open the door to new therapeutic targets for more personalized treatment strategies.

### 3.6 Redox modifications

Reactive oxygen species (ROS) and reactive nitrogen species (RNS) are signaling molecules produced by aerobic metabolism and are essential for various biological processes, as they play a central role in regulating the balance between reduction and oxidation processes at the cellular level (redox homeostasis). A key mechanism mediating ROS and RNS signaling is PTMs involving oxidation-reduction processes in proteins, known as *redox modifications*, which play an important role in cellular functions, homeostasis, and other biological processes. However, an imbalance between the production and clearance of reactive species can occur in disease states, leading to excessive levels and the generation of oxidative and nitrosative stress, which can result in aberrant redox PTMs (113–115). A key feature of many ADs is increased oxidative stress and a chronic inflammatory state, potentially leading to an excess of reactive chemical species capable of modifying the primary structure of proteins and generating neoepitopes (114, 115) (**Figure 1A**).

Virtually, all amino acid side chains can undergo redox PTMs, and numerous proteins have been shown to be modified by ROS and RNS (113, 116). Some of the most common redox PTMs include oxidation of cysteine and methionine residues, nitrosylation, glutathionylation, lipid peroxidation, and carbonylation. Redox modifications can be classified as reversible or irreversible depending on the degree of oxidative stress in the environment (113).

Increased oxidative stress and/or poor antioxidant status are involved in the pathogenesis of RA (117). Among patients with RA, ILD and cardiovascular disease, increased oxidative stress has been associated with deficiency of the antioxidant enzyme paraoxonase-1 (PON1). Moreover, osteoclasts, the cells responsible for bone erosion in RA, can increase their activity through the synthesis of ROS (118).

In the context of this inflammatory disease, several studies have found that COL2 can undergo redox modifications that trigger the synthesis and release of autoAbs against them. Immunization with hydroxyl radical-modified COL2 in rats induce significantly higher Ab titters compared to native COL2 and trigger an earlier and more severe form of arthritis (115). It has also been suggested that COL2 may undergo modifications induced by reactive radicals found in inflamed joints, generating neoantigenic epitopes (119).

Lipid peroxidation generates reactive aldehydes such as malondialdehyde (MDA), which covalently modify proteins by forming MDA-protein adducts. MDA can also react with acetaldehyde to produce highly immunogenic malondialdehyde-acetaldehyde (MAA) adducts. MAA adducts accumulate in the synovial tissue of patients with RA to a significantly greater extent than in osteoarthritis, and co-localize with citrullinated proteins within the inflamed synovium, supporting a potential role in the amplification of autoimmune responses. In a study using MAA-modified human albumin, a correlation was found between the responses of anti-MAA Ab isoforms and ACPA in patients with RA. These findings raise the possibility that MAA-modified proteins may actively contribute to the breakdown of self-tolerance, thereby driving the development of the autoimmune response. Moreover, anti-MAA was found in the serum of a significant proportion of ACPA-negative patients, suggesting potential utility as a biomarker in seronegative RA, although further studies are required to confirm this finding (120). MAA adducts have also been described in other inflammatory diseases, such as cardiovascular disease (121). Although anti-MAA antibody levels have been associated with radiographic disease progression, as well as cardiovascular and pulmonary complications and responses to biological therapies, their potential relationship with heart failure in RA has not yet been determined (122) (**Table 2**).

Another phenomenon described in the context of RA is that oxygen free radicals can alter the structure of human IgG, producing forms with increased reactivity toward RF Abs (123).

A recent study investigated modifications of carbonic anhydrase (CA) by end-products of oxidative and nitrosative stress. Modifications induced by peroxynitrite (PN) triggered an immune response against CA isoenzymes in mice. Consistent with these findings, patients with RA and Sjögren’s syndrome shown significantly higher titers of autoAbs against PN-modified CA isoenzymes compared to healthy controls (124).

Finally, both citrullination and carbamylation can be influenced by the redox environment, especially under oxidative stress or inflammatory conditions. Regarding citrullination, PAD activity is regulated by both calcium and redox conditions. A reducing environment promotes enzyme activity, while ROS inhibits it. On the other hand, carbamylated antigens are generated under oxidative conditions. According to these findings, it has been proposed that Abs against carbamylated and citrullinated Ags could serve as biomarkers reflecting the predominant redox environment in RA patients and thus could help classify patient groups and guide more specific and personalized antioxidant therapies (125).

### 3.7 Hydroxylation

*Hydroxylation* is characterized by the addition of a hydroxyl group to amino acid residues, mainly proline and lysine, in a reaction catalyzed by hydroxylases. This protein modification significantly influences various signaling pathways and other biological processes associated with metabolism and diseases such as neoplasia and autoimmunity (126, 127).

Notably, during COL2 biosynthesis, specific amino acid residues undergo hydroxylation which is essential for stabilizing the COL2 triple helix and fibrils. Therefore, altered hydroxylation could affect the normal structure of articular cartilage (39, 128). Myers et al. studied the role of COL2 PTMs by producing native COL2 with varying levels of glycosylation and hydroxylation and found that the intensity of the immune response against COL2 in CIA correlated with the level of lysine hydroxylation and glycosylation (129).

### 3.8 Deamidation

*Deamidation* involves the removal of the amide bond from the side chain of an amino acid residue, leading to the synthesis of a free carboxylic acid, converting a neutral residue into a negatively charged one and isomerizing the primary sequence. This is a common PTM in proteins and peptides, where deamidation of asparagine or glutamine residues leads to the formation of aspartic acid or glutamic acid, respectively. This process can occur spontaneously or in the case of glutamine, be catalyzed by calcium-dependent tissue transglutaminases (tTG) (130). This family of enzymes is involved in other biological functions, including cell growth and differentiation, induction of apoptosis, cell adhesion, signal transduction, receptor-mediated endocytosis, and extracellular matrix modelling (131, 132). Moreover, tTG has been implicated in the pathogenesis of inflammatory diseases such as RA. Specifically, elevated levels of anti-tTG IgG and IgA have been found in both the synovial fluid and serum of affected patients (133–135). Accordingly to these findings, Dzhambazov et al. evidenced that tTG mediates both deamidation and cross-linkink of the immunodominant T-cell epitope COL2 259–273. While T lymphocytes recognize both the native and modified versions of this peptide, the substitution of glutamine with glutamic acid at position 267 reduces MHC II binding affinity. Furthermore, *in vivo* administration of tTG increased the incidence, severity, and histopathological manifestations of CIA in murine models (131) (**Figures 1A, B**).

## 4 Potential therapeutic targets

The incidence of ADs has increased in recent decades. In RA, like in other ADs, the therapeutic goal is to achieve remission or, failing that, to attain low disease activity (136). Most currently available therapeutic options act by inducing global immunosuppression and can produce undesirable side effects, hence the growing interest among researchers in alternative treatment options with more specific mechanisms of action (136, 137). As outlined in **Figure 1C**, antigen-specific immunotherapy has been proposed in RA to restore immune tolerance to self-Ags by selectively targeting pathogenic B- or T-cell subsets, without broadly affecting systemic immunity. Some potential therapeutic targets for this disease are outlined below.

### 4.1 Tolerogenic vaccines

Tolerogenic Ag-specific vaccines have shown promising results, particularly those based on COL2 (136) (**Figure 1C**). A recent study by Jin et al. demonstrated that a vaccine targeting a citrullinated antigen (citAg) improved CIA symptoms by downregulating proinflammatory cytokines, including IL-6 and TNF-α, reducing Th1 and Th17 helper T cell subsets, and inhibiting Ag-specific memory responses (136). Furthermore, the efficacy of a RA vaccine has recently been demonstrated. This vaccine is composed of the mouse major histocompatibility complex class II (A^q^) protein conjugated to COL2_259-273_, which is galactosylated at position 264. To convert this vaccine into a therapy for RA patients and improve its efficacy, the glycine residue at position 265 was modified and the vaccine was conjugated with the human DRB1*04:01 molecule. Consequently, this modified vaccine (designated as DR4-AL179) prevented the development of RA by inducing a potent immunosuppressive response. Notably, DR4-AL179 successfully suppressed RA in mice expressing DRB1*04:01, bypassing the requirement for galactosylation at position 264 (138).

### 4.2 Regulatory T cells adoptive therapies

Regarding regulatory T cells (Tregs), lower numbers of Tregs have been observed in RA patients compared to healthy individuals, as well as impaired suppressive function, which may favor widespread activation of autoreactive T cells and systemic inflammation (139, 140). Thus, in recent years, Tregs have been used to regulate the immune response in ADs, either through adoptive cell transfer or gene therapy (141) (**Figure 1C**).

Adoptive polyclonal Tregs immunotherapy, which involves the infusion of autologous or allogeneic Tregs previously isolated, activated, and expanded *ex vivo*, has been proposed as a novel and promising therapeutic strategy for RA (140, 142). Studies in CIA models have reported that the administered Tregs homed to the synovial tissue, where they suppress T-cell proliferation ultimately leading to a significant reduction in disease severity (143). In addition, exogenous and induced Tregs demonstrated therapeutic efficacy in CIA rats by expanding endogenous Tregs while reducing B cells, Th1/Th2 ratio, and IL-6 secretion. Consequently, Treg cell transplantation represents a promising therapy for RA (144). In another study, inhibition of osteoclastogenesis and improvement of clinical symptoms in CIA were also observed after a single transfer of polyclonally activated Tregs (145). However, the use of polyclonal Tregs may induce nonspecific tolerance and increase the risk of malignancy and infections, leading some researchers to propose the use of antigen-specific regulatory T cells (140, 141).

The possibility of combining Treg therapy with other immune-modulating therapies is also being explored to achieve a more effective and comprehensive therapeutic outcome. Some immunosuppressive drugs commonly used in RA reduce Tregs proliferation and survival. Thus, combining Tregs with other cell therapy methods may produce synergistic effects in the treatment. However, such approaches are yet to be explored (146).

Tregs can be endowed with antigen specificity via transgenic TCRs or chimeric antigen receptors (CARs). TCR-engineered Tregs can recognize both surface and intracellular Ag peptides and should exhibit high affinity for MHC. While TCR-engineered Tregs have shown promise in treating RA, their clinical application is limited by the requirement to identify appropriate high-affinity interaction between the TCR and the self-peptide-MHC complex (140, 141). CAR-T cells, in contrast, are bioengineered T cells expressing specific receptors on their membranes that recognize target cell-specific Ag independently of MHC (140–142). Interestingly, *in vitro* results have been achieved using CAR-T cells targeting specific citrullinated peptide epitopes, such as citrullinated vimentin, citrullinated COL2, citrullinated fibrinogen, citrullinated tenascin-C, and cyclic citrullinated peptide, engineered from T cells derived from CIA mouse models or RA patients. These CAR-T cells not only specifically recognized and eliminated anti-COL2 antibody-secreting B cells from CIA mice but also depleted autoreactive antigen-specific B cells in RA patients (139) (**Figure 1C**).

Since circulating peripheral Tregs constitute only a small fraction of blood cells, abundant CD3+, CD4+ T cells can be isolated and transduced with *FOXP3* complementary DNA (cDNA), alongside CAR constructs targeting a specific molecule. CAR-Treg therapy has the capacity to suppress effector T cell functions, proliferation and survival, through the secretion of anti-inflammatory cytokines, suppression of antigen presenting cells (APCs) activity, IL-2 consumption and Fas/FasL-mediated apoptosis. Thus, it exerts highly specific immunomodulation within inflamed pathological tissues while preserving immune homeostasis in healthy tissues (147, 148). Accordingly, some clinical trials are investigating genetically modified Tregs to express CARs directed against inflammation-associated markers or disease-relevant autoantigens, with the goal of re-establishing immune tolerance and selectively suppressing pathogenic autoimmune responses within the inflamed joints of patients with RA. A representative example is REGULATE-RA, an ongoing first-in-human trial designed to assess the safety and tolerability of autologous Tregs engineered with a CAR construct. This therapy specifically targets citrullinated proteins localized in the inflamed synovium of patients with RA (148).

Therefore, the adoptive transfer of Tregs cells and the preservation of their regulatory functions represent a promising approach for improving the treatment of RA. CAR-Tregs cells may be a helpful tool to treat or even to cure this disease. However, a better understanding of Tregs cells and their regulatory mechanisms is necessary to improve the clinical application of Treg therapy in RA (139).

### 4.3 PAD inhibitors

As discussed previously, PADs enzymes play a critical role in RA pathogenesis. Consequently, significant efforts have been directed toward the development of PAD inhibitors as therapeutic agents. These include both irreversible and reversible inhibitors. Irreversible inhibitors covalently modify the active site cysteine residue of PAD enzymes, achieving a significant reduction in disease severity, but carry risk of off-target effects and toxicity (149, 150). In contrast, reversible inhibitors bind non-covalently to PADs enzymes, reducing the likelihood of off-target effects, albeit often at the expense of selectivity and potency (149).

Early research focused on the development of pan-PAD inhibitors capable of targeting all PAD isoenzymes. Nevertheless, more recent efforts have focused on the design of selective isoenzyme inhibitors targeting specific PAD isozymes implicated in RA pathogenesis, like PAD4 (149, 151). Current challenges in developing PAD-targeted therapies for RA include achieving adequate potency, selectivity, *in vivo* stability and bioavailability of PAD inhibitors (149) (**Figure 1C**).

### 4.4 Transglutaminases inhibitors

An increase in the expression of transglutaminase 2 (TG2) or its transglutaminase activity has been seen in numerous diseases. TG2 has been found to be upregulated in arthritic joints, and its transglutaminase activity has been strongly associated with cartilage degradation in CIA. Its inhibition may represent a promising approach for the development of therapies aimed at preventing or halting the cartilage degradation process in arthritis (152) (**Figure 1C**).

### 4.5 NETosis inhibitors

NETs play a role in the pathophysiology of several diseases, including RA, and their inhibition may present an interesting therapeutic strategy (153, 154). While further research is needed to clarify the mechanisms of NET formation in RA, some studies have found that ACPAs could serve as therapeutic agents in neutrophil-mediated inflammatory diseases. In mice with chronic arthritis, Chirivi et al. found that therapeutic ACPAs, particularly those targeting citrullinated histones H2A and H4, inhibited NET release and likely promoted their uptake by macrophages, resulting in reduced joint tissue damage (155, 156). Based on these findings, a monoclonal Ab (CIT-013) with high affinity for citrullinated histones H2A and H4, and strong anti-inflammatory effects by inhibiting NET metabolism *in vivo*, was described a few years later (154). Numerous studies, in recent years, have explored additional mechanisms for inhibiting NETosis, with some reporting conflicting results, such as those involving PAD4 inhibitors, highlighting the need for further investigation (153) (**Figure 1C**).

### 4.6 Complement inhibitors

The complement pathway plays an important role in RA pathogenesis by sustaining immune activation, NF-κB–mediated inflammatory signaling and cytokine amplification. Complement activation contributes to synovial inflammation and joint damage in RA, making the complement cascade a promising therapeutic target for this disease. Complement inhibitors, such as C3, C5 and C5a receptor antagonists, have demonstrated promising efficacy in preclinical and early clinical studies and may represent a promising therapeutic target, particularly for RA difficult-to-treat and refractory cases (157, 158) (**Figure 1C**).

## 5 Discussion

RA is a systemic autoimmune inflammatory disease characterized by chronic synovitis of joints and ultimate cartilage and bone damage. Moreover, systemic manifestations affecting other organs and tissues may be present contributing to increased morbidity and mortality. The loss of the suppressive capacity of Treg cells in the affected tissues and the existence of a pro-inflammatory chronic environment maintained by immune cells, autoantibodies and cytokines are features of this disease. Nevertheless, as other ADs, its etiopathogenesis is not fully understood and elucidating it has become a priority for many researchers.

PTMs play an important role in the early pathogenesis of RA since they have been recognized as an important source of neoantigens capable of breaking immune tolerance and promoting the development of the disease. Various modifications associated with different self-Ags have been described in RA. Despite the results obtained in several studies, further research is needed for a better understanding of mechanisms involved in its pathogenesis and to elucidate the role of PTMs in the onset and underlying mechanisms of RA.

Currently, most available therapeutic options act systemically and can produce undesirable side effects. In recent years, considerable efforts to develop alternative therapeutic strategies have been made with encouraging results. However, future investigations will help determine the utility of modified peptides for diagnosis and patient monitoring as well as identify and improve promising new therapeutic targets for more personalized and selective treatments.
